# Better together? Social distance affects joint probability discounting

**DOI:** 10.3758/s13421-022-01290-6

**Published:** 2022-03-10

**Authors:** Diana Schwenke, Ulrike Senftleben, Stefan Scherbaum

**Affiliations:** 1 Department of Psychology, Technische Universität Dresden, Dresden, Germany; 2 Aston Laboratory for Immersive Virtual Environments, Aston Institute of Health and Neurodevelopment, Aston University, Birmingham, UK

**Keywords:** Probability discounting, Joint decision-making, Social distance, Process tracing

## Abstract

Deciding together is common in our everyday life. However, the process of this joint decision-making plays out across different levels, for example language, intonation, or non-verbal behaviour. Here we focused on non-verbal interaction dynamics between two participants in probability discounting. We applied a gamified decision-making task in which participants performed a series of choices between a small but safe and a large but risky reward. In two experiments, we found that joint decision-making resulted in lower discounting and higher efficiency. In order to understand the underlying mechanisms in greater detail, we studied through which process this variation occurred and whether this process would be modulated by the social distance between both participants. Our findings suggested that socially close participants managed to reduce their discounting by interactive processes while socially distant participants were influenced by the social context itself. However, a higher level of efficiency was achieved through interactive processes for both groups. In summary, this study served as a fine-grained investigation of collaborative interaction processes and its significant impact on the outcome of choices with probabilistic consequences.

**Supplementary Information:**

The online version contains supplementary material available at 10.3758/s13421-022-01290-6.

## General introduction

“Two heads are better than one” is an often-said proverb. Hence, many important decisions in life – which are very often decisions with probabilistic outcomes, for example, whether one should take out insurance – are made together with an adviser or our partners and families rather than by a single decision-maker alone. However, combining individual opinions into a unanimous decision can be difficult. For individual decisions with probabilistic outcomes, it is well known that some people turn down a large pay-out that is uncertain in favour of a smaller but safe option, while other people make the opposite decision (Holt et al., 2003; Madden et al., 2009; Reynolds et al., 2004). This is because the extent of devaluating a value by its probability, referred to as ‘probability discounting’, is subjective. More risk-averse individuals judge a probabilistic reward as unfavourable compared to more risk-tolerant individuals – even if that option is not preferable according to a normative reference. Though we know a lot about individual probabilistic decisions, our knowledge of how such choices are made together is rather limited, which is all the more surprising in light of the fact that many such decisions are performed together in real life (e.g., when making decisions about life insurance or home insurance). Hence, we face open questions, such as how choices made by groups differ from the average preferences of their individual minds and which underlying processes and moderating factors may influence their decision-making.

In the present study, we aim to investigate those open questions in joint decision-making in probabilistic discounting. We conducted two experiments to investigate (1) whether or not joint decision-making would influence probability discounting choices, (2) how two people together resolve probabilistic trade-offs, and (3) whether the social distance between the two participants influences probabilistic discounting and the process through which joint decision-making occurs.

In Experiment 1, participants completed a series of probability discounting choices in a gamified experimental set-up that allowed us to focus on non-verbal interaction dynamics within each decision-making trial. We then conducted a second pre-registered experiment in which we manipulated the social distance between the two participants, investigating the differences between socially close and socially distant participants in terms of their discounting behaviour and the interactive processes through which they reach a mutual decision.

### Discounting decisions and social effects

When making decisions under uncertainty, people often prefer a small but safe reward over a larger but risky reward, even if the probabilistic option has a higher expected value than the safe one. This is because people tend to overweigh probabilities (Blackburn & El-Deredy, 2013; Kahneman & Tversky, 1979). However, initial evidence indicates that this pattern can be modified when decision-making is embedded in a social context.

Firstly, it has been shown that choices that were made on behalf of someone else showed a higher tendency towards the larger, riskier option and were also closer to a normative reference. Consequently, discounting was increased more for self-serving than for surrogate decisions, a pattern that occurred both in probabilistic discounting and also in delay discounting, the tendency to discount rewards if they are paid out with a temporal delay (Albrecht et al., 2011; Batteux et al., 2017; Ziegler & Tunney, 2012). Further, managing choices for others reduces loss aversion, the tendency to be more sensitive to negative outcomes and therefore to avoid losses (Andersson et al., 2014). This finding is in line with surrogate discounting under the assumption that both risk aversion in probability discounting as well as the present bias in delay discounting are driven by emotional and impulsive responses, and that these responses decline with greater social distance between the decision-maker and the recipient (Ziegler & Tunney, 2012). Further evidence for the effect of social influence on discounting choices in general showed that the observation of other people making short-term oriented delayed discounting choices increased the probability of choosing an immediate instead of a delayed option (Gilman et al., 2014). Similarly, Calluso et al. (2017) found an increase of impulsive choices for farsighted subjects after observing their opposed choice pattern trial by trial, while the reverse effect was found for impulsive participants.

Though this indicates that social factors may play a role, there are surprisingly few studies addressing the issue of joint decision-making in the context of discounting choices. Research on group discounting studied choices that participants either made for themselves or for a hypothetical group of people between whom the outcomes were shared equally. Participants in that case were more willing to wait, meaning they showed a reduced discounting rate, compared to individual outcomes (Charlton et al., 2013). Further, how individual preferences shape the decision made by the group (and vice versa) over three phases of individual-collaboration-individual discounting (Bixter et al., 2017; Bixter & Rogers, 2019) has also been studied. During the collaboration phase, groups averaged the individual preferences of their members, while individual choices in the post-collaboration phase were more similar to each other compared to pre-collaboration.. In a previous study based on a gamified setup in delay discounting (Schwenke et al., 2017), we found initial evidence for differences between individual and joint decision-making, and found that two co-acting participants chose the sooner but smaller option less often and that they chose the optimal option (according to a normative reference) more often. We further demonstrated that the dyadic variation resulted from social interchange between both co-actors and not from a general influence of the social situation itself.

In our current study, we focused on probability discounting. While probability discounting and delay discounting are at least somewhat distinct processes (Weatherly et al., 2015), both are based on a bias towards a smaller, short-term more attractive option (the small but safe option in probability discounting and the small but soon available option in delay discounting) that leads to a devaluation of a reward with increasing temporal delay or with increasing risk, respectively (for an overview, see McKerchar & Renda, 2012). The previous research indicates that this short-term bias might be modulated through social decision-making in both types of discounting, which is why we expected to replicate our previous results.

### Theoretical input from collective decision-making

In contrast to the lack of research on the effects of group decision-making in the context of discounting choices, there is an extensive literature addressing group decision-making on a variety of other decision-making tasks (Kerr & Tindale, 2004; Kugler et al., 2012). An increased decision-quality for groups (as compared to individuals) has been found in reasoning (Cooper & Kagel, 2005; Maciejovsky et al., 2013), quantity estimation (Gigone & Hastie, 1997; Laughlin et al., 2003), and perceptual discrimination tasks (Bahrami et al., 2010) (note that there are some circumstances where groups can have detrimental effects on decision-making, e.g. risk-taking in adolescents (Gardner & Steinberg, 2005; Weigard et al., 2014)). At least two classes of theories have been proposed to explain why groups often outperform their average individual members.

The so-called ‘social facilitation effect’ suggests that the individuals themselves adjust their behaviour as a consequence of the mere presence of other people. This process occurs especially in simple or well-known tasks, with the result that people improve their individual performances (Claypoole & Szalma, 2017; Uziel, 2007). Although this phenomenon was initially researched in the context of cognitive perception tasks (Cottrell et al., 1968; Henchy & Glass, 1968; Zajonc, 1965), it also applies to the field of decision-making under risk (Gardner & Steinberg, 2005; O’Brien et al., 2011) and discounting-related choices in real-world scenarios such as food choices (Herman et al., 2003; Roth et al., 2001, see for a review Herman, 2015). Similar to discounting choices, there is evidence that a socially close relationship can enhance this effect (de Castro, 1994). Based on these observations, it can be assumed that the suppression of an unwanted or unfavourable behaviour is supported by normative expectations that are derived from the social situation, which in turn exert strong effects on people’s decision-making. Therefore, the process of social facilitation may also serve as a valid explanation for why individual decision-makers adapt their discounting in a social context.

A second line of research considers additive processes to be the key element of group superiority, especially for tasks with a demonstrable correct solution (Laughlin, 1980; Laughlin & Ellis, 1986). Accordingly, groups benefit from their group members’ individual characters, which leads to a more comprehensive pool of information and cognitive resources as well as diverse areas of expertise. On this basis, group members can combine different resources (Baumann & Bonner, 2004; Hinsz, 1990), mutually correct one another (Bahrami et al., 2010), improve their individual performances through interactive group-to-individual learning (Maciejovsky et al., 2013), or distribute different task demands (Wahn et al., 2017). In sum, groups benefit from exchanging processes through inner-group interactive dynamics.

Taking together, previous research clearly demonstrates the benefits of group decision-making on several different tasks. However, it remains unclear whether collective decision-making also influences discounting choices and through which underlying mechanisms this variation may occur.

## Experiment 1

Experiment 1 implemented a novel paradigm to answer the question whether collective decision-making influences discounting choices, focussing on probabilistic decisions, and which underlying mechanism could be responsible for potential differences.

Participants in this paradigm performed a series of choices between a smaller but safe option (SS option) and a larger but risky option (LR option). The expected value of both options, determined by choice value and winning probability, allowed us to define each choice as normatively optimal or non-optimal. All choices were performed by navigating an avatar via key-presses in a virtual grassland playing field in an individual and in a joint decision-making condition. In the joint condition, each co-actor indicated their next decision step without knowing the co-actor’s preference. Within this setup, we were able to differentiate three levels of decision-making: the *individual decision* in the individual condition, *the pre-decision* as the first individual indication of preference within the joint condition, and the *dyadic decision* as the final decision within the joint condition. Since each step was indicated by a key-press, we were further able to break down the interaction sequence into separate steps in order to analyse the decision-making process.

### Question 1

We aimed to replicate our main findings from joint delay discounting (Schwenke et al., 2017) in the field of joint probability discounting. We hypothesised that participants in the joint condition would show reduced discounting and a higher level of efficiency in the joint compared to the individual condition. Since our previous study showed that the lower discounting and the higher efficacy resulted from the interaction between the two co-actors rather than the social context itself, we predicted smaller discounting in the dyadic decision compared to the individual pre-decision.

### Question 2

**Fig. 1:**
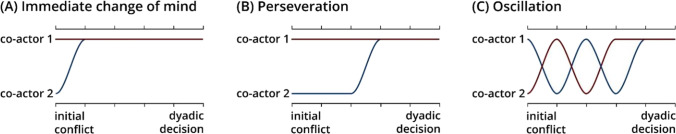
Schematic illustration of three types of conflict resolution: Immediate change of mind, perseveration, and oscillation

We aimed to study the interactive processes in greater detail. When considering potential choice patterns in the event of a conflicting pre-decision, three clearly distinguishable patterns can be derived: Immediate change of mind, perseveration, and oscillation (see Fig. 1). In order to understand why choices would deviate from a normative reference, we studied how these potential choice patterns affect the decision’s outcome in terms of discounting and efficiency.

A) *Immediate change of mind.* After opposing preferences *(initial conflict)*, one co-actor switches to the alternative option while the other co-actor repeats the prior response (switch and repetition), resulting in provisional agreement. All further decision steps should be made unanimously until the avatar reaches the option and the decision is finally made (*dyadic decision*).
B) *Perseveration.* After *initial conflict*, both co-actors repeat their prior response (repetition and repetition), resulting in continuing conflict. The conflict should be resolved by one co-actor switching to the alternative option when realising that the other co-actor will not do so (switch and repetition). All further decision steps should be made unanimously until the avatar reaches the option and the decision is finally made (*dyadic decision*).
C) *Oscillation.* After *initial conflict*, both co-actors switch to the alternative option (switch and switch), resulting in continuing conflict with reversed preferences. The conflict should be resolved by one co-actor repeating their prior decision (switch and repetition). All further decision steps should be made unanimously until the avatar reaches the option and the decision is finally made (*dyadic decision*).

### Question 3

Based on the effect of social distance on surrogate decision-making (Ziegler & Tunney, 2012), we aimed to explore whether socially close participants would differ from socially distant participants in terms of the interactive decision-making process and the decision outcome.

In addition, we ran exploratory analyses to test if the percentage of conflicting pre-decisions changed over the course of the experiment (e.g., because participants may have become better at predicting each other’s choices). However, we found no evidence for this in either experiment (see Online Supplementary Material (OSM)).

### Methods

#### Participants

Fifty-eight students of the Technische Universität (TU) Dresden, Dresden, Germany (39 females, 19 males, mean age = 23.7 years, *SD* = 4.08 years) participated in the experiment. We recruited participants from the ORSEE-based database of the Department of Psychology of the TU Dresden, Dresden, Germany (Greiner, 2015). All participants had normal or corrected-to-normal vision. Sample size was determined before any data analysis. Based on power analysis using G*Power (Faul et al., 2007), we needed a minimal sample size of 27 dyads to detect a medium effect (*d* = 0.5) found in former research (Schwenke et al., 2017) with a power of 80% (based on a paired *t*-test between choice percentages in the individual and joint condition). In the recruiting process, participants were either asked to bring a partner (close friend or partner) to do the experiment or were assigned to another participant based on their time slot preferences. Two participants who performed the experiment together were considered a dyad. This resulted in 15 dyads who were close friends or in a partnership (socially close group) and 14 dyads who did not know each other before the experiment (socially distant group). Of the dyads, there were 14 female-female, four male-male, and 11 mixed-gender dyads.

#### Apparatus

Stimuli were presented on a black background on two 19-in. CRT screens running at a resolution of 1,280 × 1,024 pixels with a 72-Hz refresh rate. For stimulus presentation we used Psychophysics Toolbox 3 in MATLAB R2010b (MathWorks Inc., Natick, MA, USA) running on two Windows XP SP2 personal computers. Participants executed their choices with the arrow keys on the keyboard of their computer while wearing noise-cancelling headphones.

#### General procedure

For the whole experiment, both participants were seated in front of two computer monitors on opposing sides of the laboratory with their backs towards each other. They were instructed to keep their eyes focused on their own screen and omit any verbal and non-verbal communication with each other. After both participants gave written consent to the experiment and provided demographic information, they were instructed by means of a standardised tutorial. Each condition (individual condition, joint condition) started with a test phase (20 trials for the first and eight trials for the second condition). Participants were told that they would be paid according to their actual choices during the experiment as a sum of all decisions across both conditions, and that they each would receive the full payment of the money they collected in the joint condition (i.e., the money was not split between the partners in the joint condition). However, since we aimed to pay a constant fee of 7.50 € to all participants, we designed the experiment so that participants’ collected values would stay below 7.50€ and we could offer them the higher, planned fee after the experiment as a better compensation.

#### Task

**Fig. 2:**
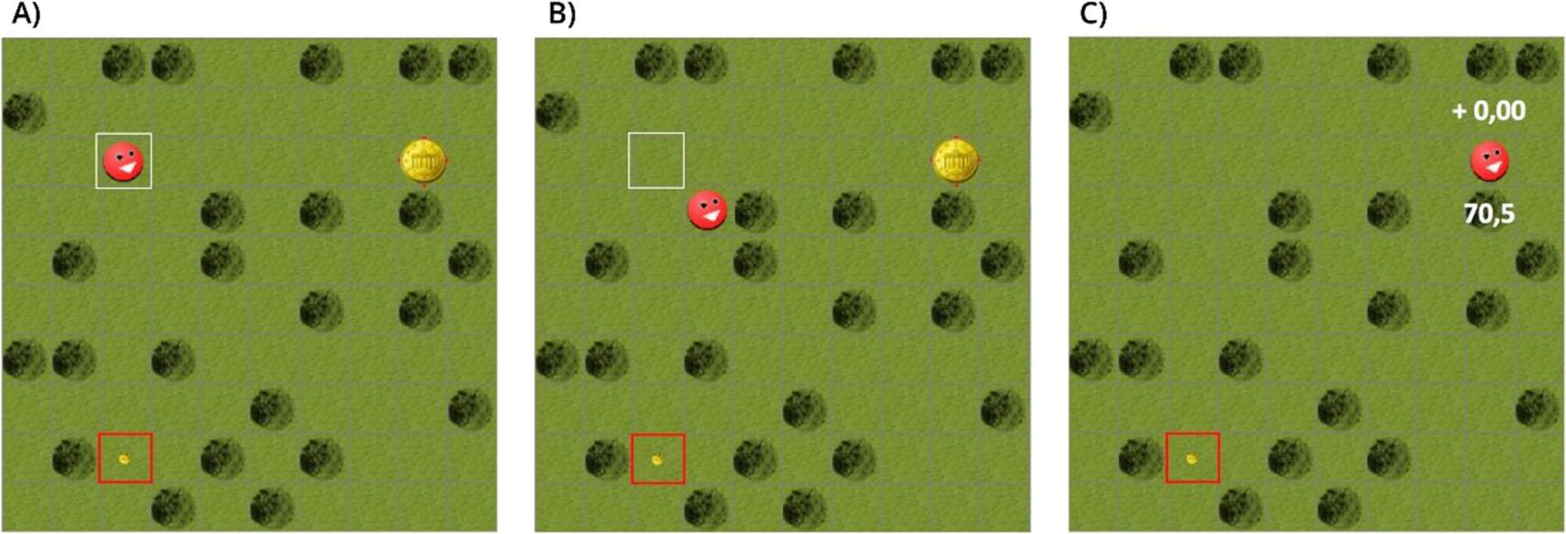
The experimental screen and procedure. **a** Each trial started with the presentation of the avatar and two coins with different values (represented by size) and different winning probabilities (represented by the length of the red border). In this example, the small coin with the complete red border had a winning probability of 100%, the larger coin had the smallest possible winning probability of 10% visualised by the four red dots. The trees (dark green) were only included for visual effects, they did not restrict movement. **b** The avatar moved after the first indication of decision-making via key-presses from both participants. Here, one co-actor preferred the save and the other preferred the risky option that led to diagonal avatar movement. **c** After the conflict was resolved, both participants collected the risky coin, which in this case they did not win

Participants’ task was to execute a sequence of choices between a safe but small (SS) or a large but risky (LR) choice option by moving an avatar via key-presses in a virtual grassland playing field (Scherbaum et al., 2016). The playing field was divided into 20 × 20 fields of 50 × 50 pixels each. The choice options were represented as two alternative coins with different values, which were illustrated by the size of the coins, and winning probabilities, which were illustrated by the length of the red border around the fields containing each coin (Fig. 2a). For a probability of 100%, the field of the coin was fully surrounded by the red frame, while for the minimal probability of 10% the frame was reduced to four red dots. For the maximum value of a choice option, the coin had the full size of the field; for the minimum value, it had the size of five pixels in diameter. Each trial started with the presentation of two coins. Participants had to collect one of the two coins by moving the avatar to the field containing the preferred choice option. The coins were placed orthogonally so that each step of the avatar would decrease its distance to one option and increase its distance to the alternative option, implying a clear decision for each movement step. Both options always had the same distance from the avatar, and this distance varied between six and eight fields across trials (see Fig. 2a). To execute a choice, participants had to move the avatar towards the preferred choice option step-by-step from one field to another via key-presses (up, down, left, right; see Fig. 2b). After the avatar reached the option, the participants were informed about whether they won the choice option and they heard a sound associated with winning or losing the coin. If they won, the value of the reward was credited to their account and they saw their current balance as well as the amount they just won. If they did not win, a ‘+0,00’ appeared above the avatar (see Fig. 2c). After an inter-trial interval of 1.3 s, the next two-choice options appeared at new positions.

**Fig. 3:**
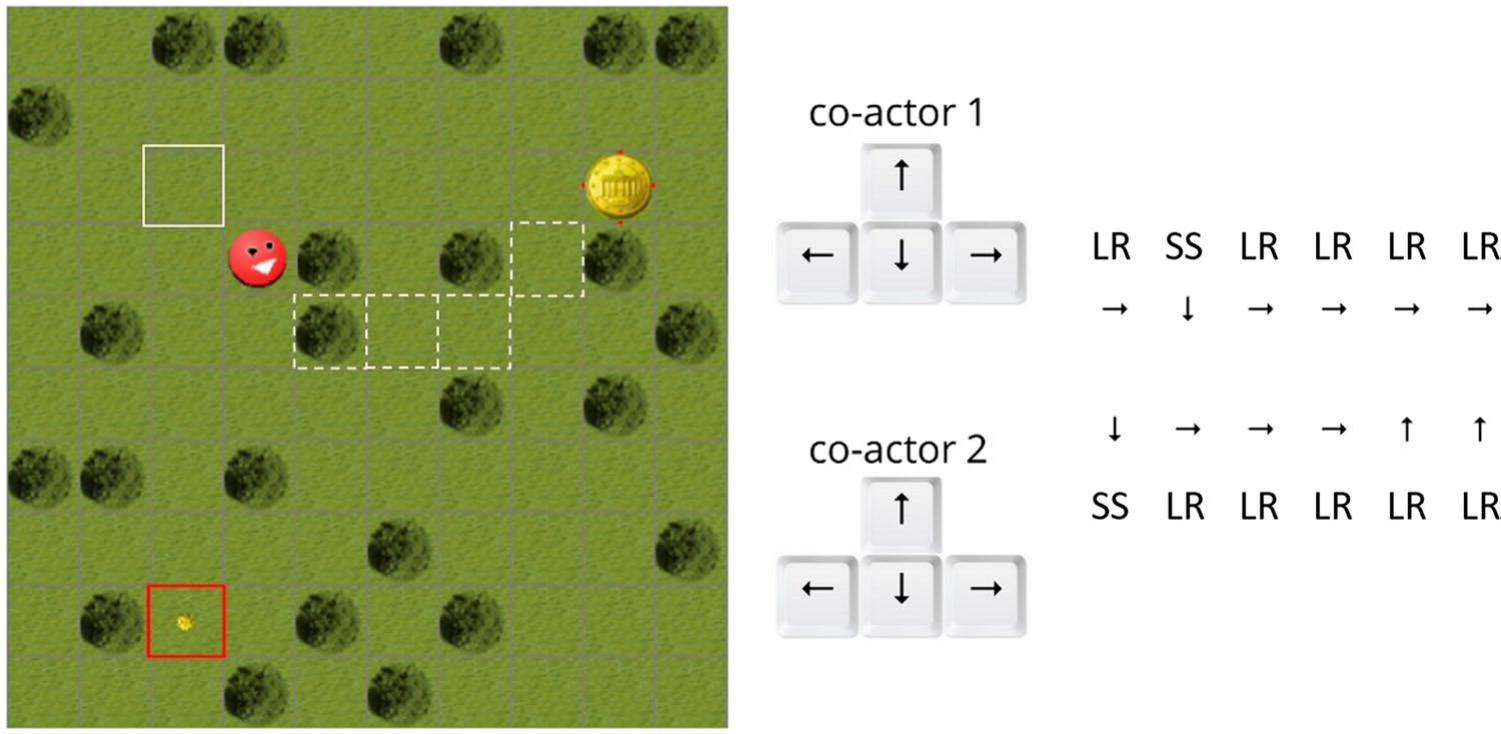
Initial dyadic conflict and conflict resolution. While co-actor 1 pressed a key (**right**) towards the large but risky option (LR), co-actor 2 pressed a key (**down**) towards the smaller but safe option (SS). Therefore, the avatar moved diagonally. After two conflicting steps, both co-actors were able to agree on the large but risky option

This procedure was performed by each participant in two conditions of decision-making: (1) In the *individual condition*, each participant individually performed the probability discounting task alone without any knowledge of her partner’s choices. Participants therefore moved the avatar step-by-step towards the preferred option by moving the avatar horizontally and vertically from field to field using the arrow keys. (2) In the *joint condition*, both co-actors performed the task together. They had to decide on a choice option together by mutually moving the avatar towards the preferred option. Crucially, the avatar started moving to another movement field only when both participants had stated their (next) preferred movement. If they both pressed the same arrow key, the avatar moved to the next field towards the chosen direction. If they pressed different arrow keys, the avatar moved to the next field in the combined direction. Hence, conflicting preferences resulted in a diagonal (indifferent) avatar movement (e.g., down and right) or no avatar movement (e.g., left and right), which was indicated by a short trembling of the avatar. In both cases, dyadic conflict led to no reduction of distance between the avatar and the coins (see Fig. 3). In this case, each co-actor had to consider what to do for the next indicated movement. They could either stay with their choice (repetition) or modify their choice (switch). Crucially, the task required both co-actors to reach unanimous consent because it was only possible to reach a coin if both co-actors moved the avatar together. Importantly, both participants were instructed to keep their eyes focused on their own screen and omit any communication.

By following this procedure, we were able to distinguish three separate levels of decision-making: (1) *the individual decision* within the individual condition by averaging both co-actors’ final decisions; (2) *the dyadic pre-decision* as the individual decision within the joint condition by averaging both co-actors’ initial keypresses towards the preferred option; (3) *the dyadic decision* as the final decision by unanimous consent.

We set no time-limit for avatar movement in the individual or the joint condition to make sure that no time pressure would impact the decision-making process or the negotiation between the two co-actors. Participants completed 180 trials in the individual and 360 trials in the joint condition. The joint condition included more trials in order to allow for further analysis of a subset of trials (i.e., trials with conflicting pre-decision).

#### Design

In each trial, a value for the LR option was randomly chosen from a set of values ranging from 65 to 85 in steps of 1. The SS value was calculated by multiplying the LR value with a value that was systematically chosen (one by one) from a set of values ranging from 0.1 to 0.9 in steps of 0.1. The SS value was then rounded to the nearest full credit, yielding a minimal value of 7 points and a maximum value of 77 points. The winning probability of the LR option was chosen systematically from a set of probabilities ranging from 10% to 90%, varying in 20% steps. The winning probability of the SS option was always 100%. The distance between the avatar and the coins was systematically varied between six and eight fields. We counterbalanced the order of the conditions of decision-making (individual-joint vs. joint-individual) across all two person-groups.

#### Statistical analysis and normative choice model

As dependent variables, we first calculated the extent of discounting by measuring the relative frequency of SS choices for each level of decision-making. Second, we calculated the decisions’ efficiency by determining participants’ relative frequency of optimal choices. Therefore, we classified each trial as an optimal or non-optimal choice according to the assumptions of a normative expected value model. With this model, we determined the optimal choice by comparing the expected values (EV) for both options to identify the option with the higher benefit:

$${\mathrm{EV}}_{\mathrm{SS}}={\mathrm{Value}}_{\mathrm{SS}}\ast 1; {\mathrm{EV}}_{\mathrm{LR}}={\mathrm{value}}_{\mathrm{LR}}\ast {\mathrm{probability}}_{\mathrm{LR}}$$

Across both conditions, in 48.37% (*SE* = 0.11%) of all trials the LR option was the optimal choice option (individual: *M* = 48.43%, *SE* = 0.14%; dyadic: *M* = 48.24%, *SE* = 0.13%). This ensured that maximising the decision efficiency (and therefore maximising the overall reward) required a careful decision-making process (instead of blindly executing one decision strategy such as always choosing the SS option). To avoid inflating statistical power, all measures for the individual decision and pre-decision were first aggregated for each individual participant and then averaged over both co-actors.

All statistical results were Greenhouse-Geisser corrected where applicable, marked with an*.

### Results

#### Hypothesis 1

**Fig. 4:**
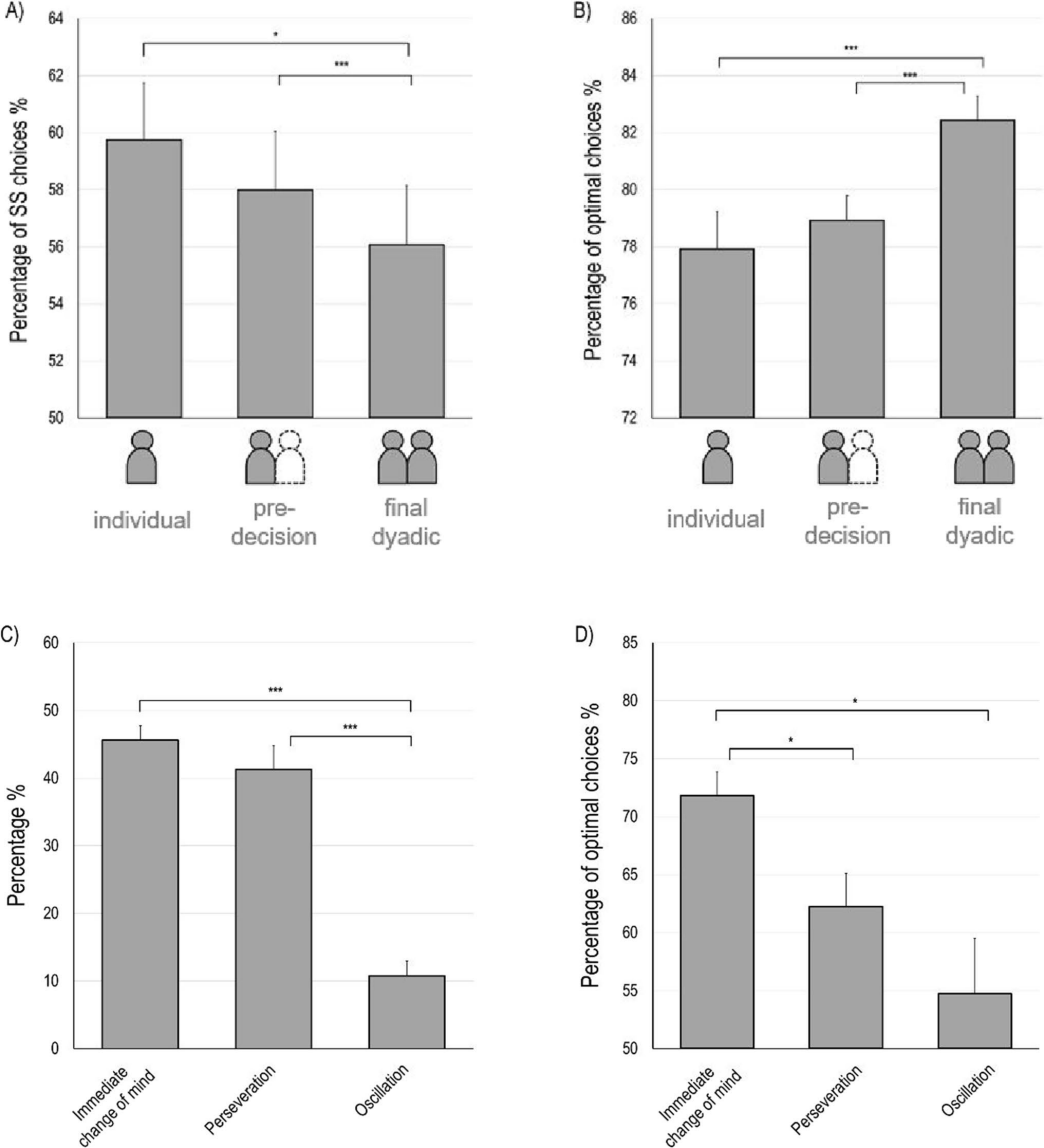
Results. **a** Average percentage of SS (smaller but safe) choices and (**b**) average percentage of optimal choices depending on the level of decision-making, from left to right: the individual decision, the pre-decision and the final dyadic decision. **c** Percentage of types of types of conflict resolution, and **d** percentage of optimal choices depending on types of conflict resolution, from left to right: immediate change of mind, perseveration, oscillation. Error bars indicate standard errors of the mean over participants. *Significance at p < .05, *** and significance at p < .001

To investigate whether dyads showed lower discounting, we calculated the relative frequency of choosing the smaller but safe (SS) instead of the larger but risky (LR) option for all three levels of decision-making (individual, pre-decision, dyadic decision). We performed planned pairwise comparisons between all three levels of decision-making and found that the dyadic decision resulted in significantly less SS choices compared to both (1) the individual decision, *t*(28) = 2.31, *p* = .028, *d* = 0.43, and (2) the pre-decision, *t*(28) = 3.54, *p* = .001, *d* = 0.66, while the difference between individual and pre-decision did not reach significance, *t*(28) = 1.07, *p* = .295 (see Fig. 4a and Table 1). To investigate whether dyads objectively improved their performance, we calculated the relative frequency of choosing the optimal instead of the non-optimal choice option for all three levels of decision-making. The optimal choice option was determined by reference to the higher expected values (choice value × winning probability) of both options. Pairwise comparison demonstrated that the dyadic decision resulted significantly more often in the optimal choice compared to both (1) the individual decision, *t*(28) = 5.07, *p* < .001, *d* = 0.94, and (2) the pre-decision, *t*(28) = 7.85, *p* < .001, *d* = 1.46, while the difference between individual and pre-decision did not reach significance, *t*(28) = 1.50, *p* = .146 (see Fig. 4b and Table 1).

**Table 1:** Descriptive statistics for hypothesis 1

		Percentage of SS choices in %	Percentage of optimal choices in %
Individual	*M*	59.75	77.92
	*SD*	10.75	7.02
Pre-decision	*M*	58.00	78.93
	*SD*	10.60	5.44
Dyadic	*M*	56.27	82.43
	*SD*	11.11	4.63

*SS* smaller but safe

Taken together, joint decision-making resulted in a lower probability of discounting and a higher level of efficiency compared to individual decision-making and the initial decision of each co-actor, as expected.

#### Hypothesis 2

To investigate the interactive processes in greater detail, we studied trials with initial conflict. Overall, 19.04% (*SD* = 7.07%) of all choices were marked as trials with opposing pre-decisions. On average, only 39.90% (*SD* = 10.38%) of these trials resulted in an SS choice, indicating that, in case of conflicting preferences, the final dyadic decision yielded less SS than LR choices, *t*(28) = 5.24, *p* < .001, *d* = 0.97 (one-sample t-test against 50%). Similarly, 66.44% (*SD* = 8.58%) of all conflict trials ended in an optimal instead of a non-optimal decision, *t*(28) = 10.32, *p* < .001, *d* = 1.92(one-sample t-test against 50%).

To understand how participants solved their initial conflict, we further classified all conflict trials by their type of conflict resolution. We therefore analysed each step towards an option for each individual co-actor in dependence of their own previous step (repetition vs. switch) and in dependence of their partner’s step (repetition vs. switch), resulting in either a continuing conflict or in provisional agreement. Following this procedure, all conflict trials could be classified as one of the following three distinct types of conflict resolution. (A) *Immediate change of mind* included trials where, after initial conflict, one co-actor switched to the alternative option while the other co-actor repeated the prior choice. (B) *Perseveration* included trials where, after initial conflict, both co-actors repeated their prior choices. (C) *Oscillation* included trials where, after initial conflict, both co-actors switched to the alternative option. Because we did not formulate specific a priori hypotheses to predict specific differences, we conducted a repeated-measures analysis of variance (ANOVA) to study if any type of conflict resolution occurred more frequently than another and found a significant main effect, *F*(1.39, 39.05) = 33.76, *p* < .001, *η*_*p*_^*2*^ *=* 0.55*. We then performed post hoc comparison *t*-tests and found a higher relative frequency of immediate change of mind than oscillation, *t*(28) = 12.91, *p* < .001, *d* = 2.40, and a higher relative frequency of perseveration than oscillation, *t*(28) = 5.69, *p* < .001, *d* = 1.06, but no significant difference between the relative frequency of immediate change of mind and perseveration, *t*(28) = 0.82, *p* = .420 (see Fig. 4c and Table 2). We further analysed in what way the type of conflict resolution was related to the decision’s outcome. We performed a repeated-measures ANOVA of the relative frequency of SS choices (respectively optimal choices) depending on the type of conflict resolution. We found no significant difference for the relative frequency of SS choices, *F*(1.53, 35.13) = 1.11, *p* = 0.326*, but a significant main effect for the relative frequency of optimal choices, *F*(1.39, 31.91) = 4.72, *p* = .027, *η*_*p*_^*2*^ *=* 0.17*. Post hoc comparison revealed that dyads showed a higher relative frequency of optimal choices when conflicts were solved as immediate change of mind compared to both perseveration, *t*(28) = 3.33, *p* = .002, *d* = 0.62, and oscillation, *t*(23) = 2.66, *p* = .014, *d* = 0.54. We found no significant difference between perseveration and oscillation, *t*(23) = 1.36, *p* = .189. Hence, a quick resolution of conflict improved decision-making more than perseverative and oscillatory conflict resolution (see Fig. 4d and Table 2).

**Table 2:** Descriptive statistics for hypothesis 2

		Percentage of conflict trials in %	Percentage of optimal choices in %
Immediate change of mind	*M*	45.61	71.81
	*SD*	11.43	10.86
Perseveration	*M*	41.28	62.27
	*SD*	18.79	15.23
Oscillation	*M*	10.79	54.73
	*SD*	11.76	23.39

#### Hypothesis 3

As an exploratory analysis, we tested whether the social distance between the two participants had any impact on the decision-making process and hence the decision outcome. We performed repeated-measures ANOVAs on the relative frequency of SS choices and on the relative frequency of optimal choices (see Table 3 for descriptive statistics). For optimal choices, we found a significant interaction effect between level of decision-making and social distance, *F*(2,54) = 5.52, *p* = .007, *η*_*p*_^*2*^ = 0.17; all other effects were not significant (see OSM). Specifically, socially distant dyads already showed a change in decision-making in the pre-decision of the joint condition (i.e., more optimal choices as compared to the individual condition) with further improvement in the dyadic decisions, whereas socially close dyads only showed improved decision-making in the dyadic decisions. Hence, the social distance between both participants influenced the decision-making to some extent.

**Table 3:** Descriptive statistics

		Percentage of SS choices in %		Percentage of optimal choices in %	
		Close	Distant	Close	Distant
Individual	*M*	59.77	59.73	78.45	77.34
	*SD*	9.13	12.62	7.30	6.94
Pre-decision	*M*	60.03	55.81	77.91	80.01
	*SD*	12.33	8.26	5.45	5.40
Dyadic	*M*	57.80	54.63	81.00	83.96
	*SD*	12.77	9.20	4.70	4.19

*SS* smaller but safe

### Discussion

In Experiment 1, we studied the potential influence of joint decision-making on probabilistic discounting. In accordance with our prediction, we found that decision-making was improved in the joint condition: dyads chose the SS option less often and the optimal option more often compared to their average individual decision-making. As expected, we found no significant differences between the pre-decision and the individual decision, but fewer SS and more optimal choices in the final dyadic decision compared to the pre-decision. This indicates that dyads benefited from the interactive collaboration between the two co-actors. This replicated the exact behavioural pattern we previously found in dyadic delay discounting (Schwenke et al., 2017).

In order to gain more insight into these interactive dynamics, we further identified three different patterns of interaction that occurred during the process of resolving conflicting preferences: immediate change of mind and perseveration as the two most common patterns, and oscillation as a less frequent pattern. Importantly, immediate change of mind led to a significantly higher relative frequency of optimal choices compared to perseveration and oscillation.

Finally, we found initial evidence for an effect of social distance on collective probability discounting, but the results were inconclusive and the sample too small to reach a reliable interpretation. For that reason, we aimed to study this possible difference between socially close and socially distant co-actors in a preregistered study (Experiment 2).

## Experiment 2

Studies on surrogate discounting have demonstrated that the decision’s recipient can play a central role since choices differ depending on whether the decision-maker herself or another person is the recipient (Albrecht et al., 2011; Batteux et al., 2017; Ziegler & Tunney, 2012). The identity of ‘the other person’ and the social distance between the decision-maker and the recipient also seems to be crucial. Ziegler and Tunney (2012) found that discounting functions for self-serving choices were more similar to those for hypothetical first-degree relatives (parents and sibling) compared to more distant relatives and complete strangers. Similarly, Batteux et al. (2017) found that probability discounting rates were reduced (i.e., the smaller but safe option was chosen less often) when deciding for a stranger as compared to a friend or oneself. This pattern is in line with research showing that participants underestimate how much their own preferences can change in the distant future. This so-called ‘presentism bias’ applied to their own future selves and to those of close others (Renoult et al., 2016), but was not applicable to distant strangers (Bauckham et al., 2019; Pronin et al., 2008). One explanation for this pattern is a variance in empathy (O’Connell et al., 2013). People tend to empathise less when reasoning about strangers and thus think in a more rational way due to the benefit of a psychological distance from their own emotional impulses (Lee & Atance, 2016). If in the reverse case people feel close to somebody, they automatically simulate their own internal states and respond more to the emotional aspects of decision-making (Bechara & Damasio, 2005; Loewenstein, 1996). However, the literature on the differential impact of social distance on surrogate decision-making is still sparse and shows ambiguous results (Montinari & Rancan, 2018). It remains unclear whether the same effects apply for joint decisions. For that reason, we aimed to study the potential effect of social distance on probability discounting choices in Experiment 2.

### Aim and research question

Experiment 2 was a preregistered study (osf.io/ea5qg). We aimed to replicate Experiment 1 and to systematically study the influence of social distance on probability discounting with a sufficiently large sample. Two groups of participants were compared: 30 pairs of participants who did not know each other prior to the experiment (socially distant) and 30 pairs of participants who were best friends or partners (socially close). We aimed to study the following research hypotheses.

#### Hypothesis 1

We expected to replicate Experiment 1’s general effect of smaller discounting in the joint condition compared to the individual condition. We further hypothesised that socially distant participants would show a different decision-making pattern than socially close participants. Specifically, we hypothesised that socially distant (but not socially close) participants would show reduced discounting in their pre-decision compared to their individual decision-making (indicating the effect of social facilitation). Further, we expected smaller joint discounting compared to participants’ pre-decisions (indicating the effect of social collaboration) for both groups.

#### Hypothesis 2

We expected a higher level of efficiency for the joint condition compared to the individual condition for both groups. For the difference between the individual decision and the pre-decision, we expected a smaller difference for socially close co-actors compared to socially distant co-actors. In contrast, for the difference between pre-decision and final decision, we expected no systematic difference between socially close and distant co-actors.

### Methods

#### Participants

We recruited participants from the ORSEE-based database of the Department of Psychology of the TU Dresden, Dresden, Germany (Greiner, 2015). In the recruiting process, we randomly invited individuals to either participate in the study with a close friend or their partner (socially close group; *N* = 60, 39 females; mean age = 22.23 years, *SD* = 2.53 years) or to participate with a stranger (socially distant group; *N* = 60, 41 females, mean age = 22.05 years, *SD* = 2.92 years). Participants in the socially distant group were grouped based on their personal time preference. In total, 120 undergraduate students participated for partial fulfilment of course credit or €7.50. All participants had normal or corrected-to-normal vision. Sample size was determined before any data analysis. Based on power analysis using G*Power (Faul et al., 2007), we needed a sample size of 30 two-person groups for each condition (yielding a total sample of *N* = 120 participants) to detect a medium effect (*d* = 0.65) in an unpaired *t*-test with a power of 80%. The effect size was based on our results in Experiment 1, where the increase in optimal choice percentage from the individual condition to the joint condition was larger for socially distant participants with an effect size of *d* = .9. As effect sizes are often overestimated with smaller sample sizes, we used the more conservative estimate of *d* = .65 for our power analysis. The complete data set was collected gradually on the following terms: Participants were excluded if their discounting in the individual condition was either too strong (more than 80% SS choices) or too weak (less than 20% SS choices) to prevent ceiling or floor effects in the individual condition. This was to allow any modulation in the participants’ choice behaviour due to the experimental manipulation and to ensure that we did not produce any artificial effect due to regression to the mean. To this end, we excluded six participants (five dyads) with a relative frequency of sooner smaller (SS) choices over 80%.^1^ Data collection was stopped after the final sample size of 120 valid data sets was reached.

For a description of the apparatus, general procedure, task, design and statistical analysis see Experiment 1.

### Results

#### Confirmatory analyses

##### Hypothesis 1

**Fig. 5:**
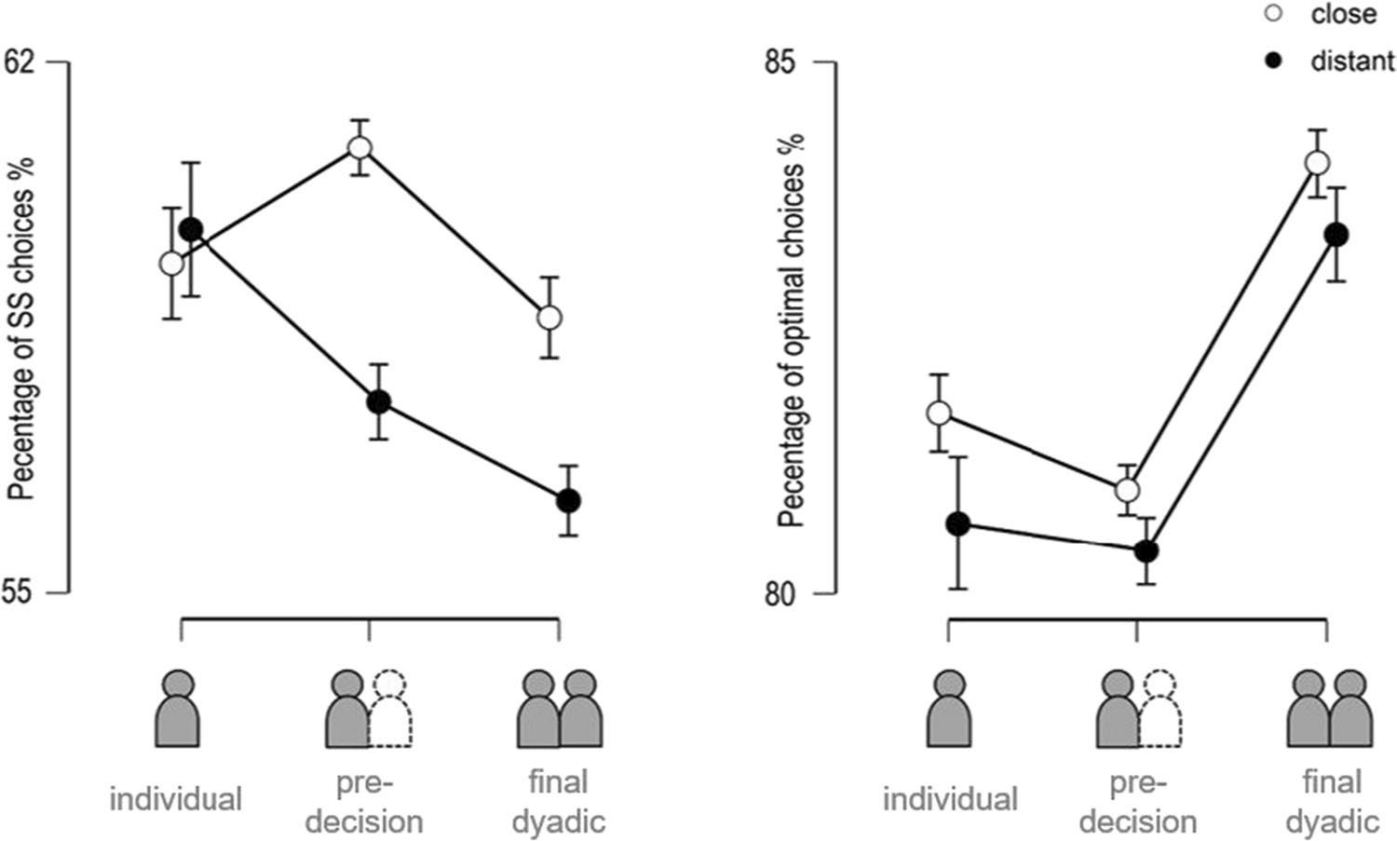
Average percentage of SS (smaller but safe) choices and average relative frequency of optimal choices depending on the level of decision-making, i.e., the individual decision, the pre-decision and the finial common decision and social distance. Error bars indicate standard errors of the mean over participants

First, we investigated whether socially close and socially distant participants decided differently in terms of the relative frequency of choosing the smaller but safe (SS) instead of the larger but risky (LR) option. We performed a repeated-measures ANOVA with the within-factor *level of decision-making* (individual, pre-decision, dyadic decision) and the between-factor *social distance* (socially close vs. socially distant) on the percentage of SS choices, and found a significant main effect for *level of decision-making*, *F*(1.22, 70.57) = 7.22, *p* =.006, *ηp*^*2*^ = 0.11*, no significant main effect for *social distance F*(1, 58) = 0.75, *p* =.391, and a significant interaction between both factors, *F*(1.22, 70.57) = 5.39, *p* =.017, *ηp*^*2*^ = 0.09 (see Table 4 for descriptive statistics). For post hoc comparison, we performed separate analyses for each group. We performed paired *t*-tests for all three levels of decision-making and found that socially close participants in the dyadic decision showed significantly smaller discounting compared to their pre-decision, *t*(29) = 4.95, *p* < .001, *d* = 0.90, while no other comparison reached significance, all *t*s < 1.83, all *p*s > .078. In contrast, socially distant participants showed significantly smaller discounting in their dyadic decision compared to both the individual decision, *t*(29) = 3.33, *p* = .002, *d* = 0.61, and the pre-decision, *t*(29) = 4.40, *p* < .001, *d* = 0.80. Importantly, socially distant participants also showed significantly smaller discounting in their pre-decision compared to their individual decision, *t*(29) = 2.06, *p* = .049, *d* = 0.38 (see Fig. 5; see Table 4 for descriptive statistics). Taken together, joint decision-making resulted in lower probability discounting regardless of the social distance between both participants, as expected. Further, we were able to confirm our hypothesis that socially distant participants already adapted their choices in their pre-decision, while socially close participants modified their choices through interaction processes.

**Table 4:** Descriptive statistics

		Percentage of SS choices in %		Percentage of optimal choices in %	
		Close	Distant	Close	Distant
Individual	*M*	59.34	59.78	81.70	80.67
	*SD*	6.70	7.22	3.83	4.45
Pre-decision	*M*	60.86	57.53	80.98	80.41
	*SD*	7.08	10.42	3.56	3.66
Dyadic	*M*	58.63	56.23	84.04	83.37
	*SD*	7.90	9.96	4.31	4.00

*SS* smaller but safe

##### Hypothesis 2

Next, we investigated whether socially close and socially distant participants decided differently in terms of the relative frequency of optimal choices. We performed a repeated-measures ANOVA with the within-factor *level of decision-making* (individual, pre-decision, dyadic decision) and the between-factor *social distance* (socially close vs. socially distant) on the percentage of optimal choices. We found a significant main effect for *level of decision-making*, *F*(1.40, 81.25) = 32.30, *p* < .001, *η*_*p*_^*2*^ = 0.36*, no significant main effect for *social distance F*(1, 58) = 0.69, *p* =.410, and no significant interaction between the two factors, *F*(1.40, 81.25) = 0.18, *p* = .755. Because we found no indication for an influence of the social distance, we performed a post hoc paired t-test between all *levels of decision-making* (across both groups) and found that participants in the dyadic decision chose the optimal option significantly more often compared to the individual decision, *t*(59) = 5.12, *p* < .001, *d* = 0.66, and the pre-decision, *t*(59) = 12.00, *p* < .001, *d* = 1.55, while the difference between individual condition and the pre-decision did not reach significance, *t*(59) = 1.18, *p* = 0.242 (see Fig. 5 and Table 4). Although this finding confirmed our hypotheses that joint decision-making would result in more optimal choices, we found no indications for any differential impact of the social distance between the two participants.

Taken together, the social distance between the two co-actors influenced the relative frequency of SS choices, as expected, but not the relative frequency of optimal choices.

#### Exploratory analyses

We analysed whether the social distance had any effect on interaction patterns between both participants. Overall, 17.29% (*SE* = 0.63%) of all choices were marked as trials with conflicting pre-decisions. On average, only 41.43% (*SE* = 1.41%) of these trials resulted in an SS choice, indicating that, in case of conflicting preferences, the final dyadic decision yielded less SS than LR choices, *t*(59) = 6.08, *p* < .001, *d* = 0.78 (one-sample t-test against 50%). Similarly, 65.48% (*SE* = 1.11%) of all conflict trials ended in an optimal instead of a non-optimal decision, *t*(59) = 14.00, *p* < .001, *d* = 1.81 (one-sample t-test against 50%). We then performed a mixed ANOVA on the relative frequency of *types of conflict resolution* (immediate change of mind, perseveration, oscillation) 2 and the between-factor *social distance*, and found a significant main effect for types of conflict resolution, *F*(1.31, 76.07) = 70.11, *p* < .001, *ηp*^*2*^ = 0.55*, but no significant effect for social distance and no significant interaction, all *F* < 0.95 and all *p* < 0.358. We then performed post hoc comparison *t*-tests and found a higher relative frequency of immediate change of mind than oscillation, *t*(59) = 19.28, *p* < .001, *d* = 2.49, a higher relative frequency of perseveration than oscillation, *t*(59) = 8.32, *p* < .001, *d* = 1.08, but no significant difference between the relative frequency of immediate change and perseveration, *t*(59) = 0.71, *p* = 0.48 (see Table 5).

**Table 5:** Descriptive statistics for conflict trials

		Percentage of conflict trials in %	Percentage of optimal choices in %
Immediate change of mind	*M*	44.34	70.05
	*SD*	10.75	11.55
Perseveration	*M*	41.86	62.90
	*SD*	17.98	15.58
Oscillation	*M*	11.63	50.71
	*SD*	11.44	28.37

We further analysed in what way the type of conflict resolution was related to the decision outcomes, similar to Experiment 1. We performed a mixed ANOVA of the relative frequency of SS choices (respectively optimal choices) depending on the *type of conflict resolution* and the between-factor *social distance*. For the relative frequency of SS choices, no effect reached significance, all *F <* 3.39, all *p* > .071*. For the relative frequency of optimal choices, we found a main effect for type of conflict resolution, *F*(1.50,78.13) = 11.72, *p <* .001*, *η*_*p*_^*2*^ *=* 0.18*. No other effect reached significance, all *F <* 2.80, all *p* > 0.082*. Similar to Experiment 1, pairwise comparisons revealed that dyads showed a higher relative frequency of optimal choices when conflicts were solved as immediate change of mind compared to both perseveration, *t*(59) = 2.96, *p* = .004, *d* = 0.38, and oscillation, *t*(53) = 4.21, *p* < .001, *d* = 0.57 (see Table 5).. The relative frequency of optimal choices was higher when conflicts were solved as perseveration compared to oscillation, *t*(53) = 2.74, *p* = .008, *d* = 0.37.

### Discussion

In Experiment 2, we followed a preregistered protocol to test whether the social distance between the two participants had any influence on joint decision-making in probability discounting.

As expected, we found that the socially close and socially distant participants showed different decision-making in terms of their discounting and the process of reaching unanimous consent. When making joint decisions, socially distant participants already showed reduced discounting in their pre-decision in comparison to their individual choices, while no such difference was found for socially close participants. This indicates that socially distant participants adjusted their personal preferences earlier than socially close participants in order to adapt to the other person. Further, both groups showed reduced discounting in their dyadic decision compared to their pre-decision, as expected. However, against our predictions, we found no systematic difference between the two groups in terms of the relative frequency of optimal choices.

In terms of the interactive decision-making patterns between the two participants, we were able to replicate our findings from Experiment 1 to some extent, but found no evidence for a differential effect of social distance.

## General discussion

Decisions in everyday life are often characterised by alternatives with different probabilities of occurrence. Given that we often make such choices together with others rather than alone, we here conducted two experiments to study the potential influence of joint decision-making on probabilistic discounting and the underlying interaction processes. We further studied whether the social distance between two co-acting participants influenced those choices, in particular whether socially close and socially distant participants employ different collaborative processes in order to reach a joint decision. In Experiment 1, we tested 29 pairs of participants and studied the social distance only as a control variable. Based on these findings, we then conducted a preregistered Experiment 2 with a sufficiently large sample including 30 pairs of participants who were socially close (partners or close friends) and 30 pairs of participants who were socially distant (participants who did not know each other before the experiment).

In both experiments, participants performed a series of choices between a smaller but safe (SS) option and a larger but risky (LR) option via a sequence of key-presses. By reducing communication to non-verbal interactive coordination, we were able to break down the interaction sequence into analysable steps, starting with the very first indication of preference of each individual co-actor within the joint condition (pre-decision) to a gradual solution of possible conflicting preferences (dyadic decision). With this we were able to analyse the specific underlying mechanisms through which probabilistic trade-offs were resolved following two major procedures. First, we compared all three levels of decision-making (individual decision, pre-decision, final dyadic decision). If the mere presence of another co-actor had any impact on the participants’ decision-making, we should have found a significant difference between the individual decision and the pre-decision. Conversely, if the joint decision emerged as a consequence of the interaction process between the two co-actors, we should have found a significant difference between the pre-decision and the dyadic decision. To gain deeper insight, we then identified three different patterns of interaction that occurred during the process of resolving conflicting preferences: *Immediate change of mind* characterised trials where one co-actor agreed to change her opinion while the other stayed with her initial choice. *Perseveration* included trials where both co-actors repeatedly preferred their initial choice, resulting in continuing conflict. In contrast, *oscillation* included trials where both co-actors switched to the alternative option, which resulted in continuing conflict with reversed preferences.

### Effects of joint decision-making on probability discounting

In Experiment 1, we found that joint decision-making resulted in less discounting and a higher level of efficiency compared to individual decision-making. This modulation was caused by the interaction between both co-actors rather than the social context itself, as expected. These findings indicate that dyads successfully managed to reduce their discounting and to improve their decision-making in terms of a normative reference. Importantly, participants always received the rewards they won in full, even in the joint condition (i.e., they did not split the payment with their partner in the joint condition). Hence, differences between joint and individual decision-making do not simply reflect an effect of reward magnitude. These findings are in line with research on group decision-making (for a review, see Kerr & Tindale, 2004; Kugler et al., 2012), and also replicate our previous findings from joint delay discounting (Schwenke et al., 2017). Furthermore, our results from both experiments demonstrated that in case of opposing preferences, the final dyadic decision yielded LR more often than SS choices and optimal more often than non-optimal choices. This contradicts the potential assumption that conflicting preferences converge randomly, because this should have led to a SS:LR ratio of 50:50. Instead, this suggests an interactive error-adjusting-process, which is also supported by the analyses of the patterns of conflict resolution. In both experiments, *immediate changes of mind* resulted in more optimal choices than *perseveration* and *oscillation.* This suggests that co-actors who initially chose the non-optimal option reassessed their preference and changed it quickly after the indication of conflict. Perseveration, in contrast, indicates that both co-actors stuck to their initial preferences, convinced that their choice was the better decision. Oscillation reveals the opposite pattern, though with the same outcome: Co-actors were generally not as convinced that their choices were superior, and consequently agreed to reassess their preferences–however, since both co-actors changed their mind, this led to ongoing conflict until one co-actor abandoned her choice. We found no difference in the relative frequency of optimal choices for *perseveration* and *oscillation,* which leads to the assumption that both a mutual overconfidence and a mutual lack of confidence result in the same decision outcome eventually.

### Effects of social distance

In Experiment 1, we found initial evidence that socially close and socially distant participants used different processes to resolve probabilistic trade-offs, but the sample was too small for any reliable interpretations. Importantly, our findings from Experiment 2 clearly confirmed such differential effects. Whereas socially close participants showed no reduced discounting in their pre-decision, socially distant participants already adapted their decision-making within their pre-decisions and chose fewer SS choices compared to their individual choices. This means that participants who decided together with a socially distant partner made more risky choices. One explanation for this finding comes from research on social distance and surrogate decision-making. Here, self-serving choices and choices on behalf of someone else were more similar in case of a close relationship (Batteux et al., 2017; O’Connell et al., 2013; Ziegler & Tunney, 2012). As Batteux et al. (2017) argue, this is because people are more affected by the outcome of a decision that they make on behalf of a friend rather than on behalf of a stranger. Hence, they are not willing to take on more risks when deciding for a friend, but decisions made on behalf of a stranger are less affected by this risk aversion. This could explain why the participants in socially distant dyads adapted their decision style to be less risk averse. Surrogate decision-making in those studies is, of course, not directly equivalent to the idea of the pre-decision in our experiments. While in surrogate decision-making, choices solely serve someone else, the pre-decision in our study serves both the decision-maker herself and the other socially close or distant participant. However, we find both concepts comparable in the sense that, in both cases, the recipient of choices is not restricted to the decision-maker but extended to a social dimension. Interestingly, social distance did not affect the relative frequency of optimal choices, indicating the two measures – the relative frequency of SS choices and the optimal choices – are separate measures that both capture the outcome of a decision, but can be modulated by different factors.

It is interesting to consider the role of trust and perceived reliability or expertise in the socially close and socially distant groups. It seems plausible that participants in socially close dyads may have increased trust in each other and may have viewed each other as more reliable, whereas participants in socially distant dyads did not know if their partner was reliable or not. This lack of trust might affect decision-making, for example by making participants more cautious. However, our results show that participants in the distant group already changed their decision-making towards the more risky option in the pre-decision. Therefore, the presence of a socially distant partner did not make them more cautious in their decisions. This raises the question of what role trust or perceived reliability plays in this type of joint decision-making.

### Limitations

In view of existing research on delay and probability discounting, our paradigm clearly differs from classic discounting research. Participants decided between choice options with relatively small choice reward values and experienced real consequences of winning or losing an option on a trial-by-trial basis (in contrast to deciding between hypothetical rewards). Despite these alterations, we argue that our findings are comparable to discounting shown in standard discounting approaches. Discounting behaviour – in the sense of devaluating a target object by time or risk – occurs in a variety of methodical procedures, for example, diverse time scales (Gregorios-Pippas et al., 2009; Kirby et al., 1999; Read et al., 2005), different forms of presenting time information (Read et al., 2005), trial-by-trial experience (Lane et al., 2003) or time ambiguity (Ikink et al., 2018). Previous research suggests there is no systematic difference in behaviour when it comes to real versus hypothetical types of rewards (Lagorio & Madden, 2005; Madden et al., 2003). Furthermore, discounting itself is not limited to monetary choices since it also occurs in choices with primary items like food or alcohol (McClure et al., 2007; Odum et al., 2006; Stillman et al., 2017). Even if these circumstances influenced discounting per se, this would not necessarily affect the phenomenon we were interested in here, namely whether decision outcomes of deciding individually or together differed and how the negotiation process influenced these decisions. However, this reasoning leads to another limitation: the level of interaction we focused on. With this work, we aimed to study non-verbal interaction dynamics among two co-actors who mutually regulated an avatar via key-presses. However, the process of joint decision-making is situated on a variety of different behavioural and cognitive levels, for example verbal participation and gestures (Maricchiolo et al., 2011), eye movement (Peshkovskaya et al., 2017), the extent of cooperation (Evans et al., 2015; Ponti & Rodriguez-Lara, 2015), or more conceptual levels such as shared cognition (Cooke et al., 2013) and alignment (Gallotti et al., 2017). This demonstrates that the combination of many different approaches is necessary in order to study social interaction in greater detail (Abney et al., 2014). Here, we focused on joint decision-making on a non-verbal level as one approach among many others. However, the fact that our main finding – improved decision quality in the joint decision – is in line with the general and higher level results from group decision-making indicates that we captured parts of the essence of the interactive decision processes in dyads.

Another potential limitation concerns the timescale of the decisions in our paradigm. Participants were quicker to navigate towards their chosen option in the individual condition than in the joint condition. Therefore, the overall time between presentations of the options and receipt (or loss) of the reward was longer in the joint condition. This additional delay could potentially affect how participants evaluate the reward options, as delays can change discounting and lead to more optimal decisions (Scherbaum et al., 2018). However, the difference in timescales was small (under 2 s on average) and the effect of social distance cannot be explained by this delay. Therefore, we do not believe that a potential delay discounting effect played a major role in our experiment.

For the social distance effect, one potential limitation is the fact that we only recruited participants through the online database of the TU Dresden Psychology Department. Therefore, participants in the distant group might still feel somewhat connected or similar to each other based on living in the same area and being part of the same university. Hence, the distance between our two levels of social distance might not have been substantial enough. In the future, social distance could be increased by recruiting participants through more diverse channels or from different areas of Germany.

### Conclusion

Making decisions together is said to make decisions better. Here, we performed two studies about joint probability discounting decisions that replicated and added to our recent study on delay discounting decisions (Schwenke et al., 2017). We showed how probability discounting decisions can benefit from collaborative interaction. Most importantly, we showed that conflicting preferences are the core element to initiate an act of communication that, as minimal as it might be, leads to a better decision outcome, and that this act of communication is relied on especially when participants are close to each other. Hence, two heads are not only better than one, but it is two people disagreeing that brings out the best of them.

## Supplementary Information

ESM 1 (PDF 128 kb)

## Footnotes

1 These exclusion criteria were chosen based on pilot studies and were preregistered before data collection began. When including all dyads in our analyses, there was no qualitative change for the optimal choice effect. For the SS choice effect, all results were qualitatively the same except for a significant main effect of social distance, *F*(1,63) = 4.06, *p* = .048, *ηp*^*2*^ = .06 (which did not reach significance when excluding the outliers). The complete data set and additional analyses scripts are available at https://osf.io/gufr6/.

2 Six out of 60 dyads did not show any oscillation at all.

## Declarations

### Open practices and data statement

The datasets generated and analysed during the current study are available in the Open Science Framework repository, https://osf.io/gufr6/. Data processing was carried out in Matlab R2015a and statistical testing was carried out using JASP 0.9.1.0 (JASP Team, 2018); all respective scripts can also be downloaded at https://osf.io/gufr6/. Experiment 2 was preregistered at https://osf.io/ea5qg.
