# Supplementary material for: Better together? Social distance affects joint probability discounting
Source: Mem Cognit. 2022 Mar 10;50(7):1513–29. doi: 10.3758/s13421-022-01290-6 (PMC9508051; doi:10.3758/s13421-022-01290-6)
Supplement: Supplementary file 1 — (PDF 128 kb) [file 13421_2022_1290_MOESM1_ESM.pdf]

Better together? Social distance affects joint probability discounting

—Supplementary materials—

Authors: Diana Schwenke, Ulrike Senftleben, Stefan Scherbaum

Exploratory analysis: Time course of conflicting pre-decisions

As a further exploratory analysis, we asked if the percentage of conflicting pre-decisions changed over the course of the experiment (for example because participants may have become better at predicting each other's choice preferences), and if this was different for socially close and socially distant dyads. We tested this in each experiment by splitting the trials of the joint condition in half and running a mixed ANOVA on the percentage of conflicting pre-decisions with the within-subjects factor Time (1st half of trials, 2nd half of trials) and the between-subjects factor Social distance (close, distant). None of the effects was significant (see Table S 1). Therefore, it seems that the percentage of conflict trials did not change over the course of the experiment and was not affected by social distance.

Table S 1

	Experiment 1		Experiment 2	
	F	*p*	*F*	*p*
Time	$F(1,27) = 0.32$	.577	$F(1,58) = 1.02$	.316
Social distance	$F(1,27) = 1.00$	.327	$F(1,58) = 1.99$	.164
Time x Social distance	$F(1,27) = 0.71$	.409	$F(1,58) = 0.96$	.332

Exploratory analysis: Effect of social distance in Experiment 1

We performed a repeated-measure ANOVA on the relative frequency of SS choices with the factor Level of decision-making (individual decision, pre-decision, dyadic decision) and the between-factor Social distance (close, distant). We found no significant main effect for social distance, $F(1,27) = 0.43$, $p = .516$, nor for the interaction between level of decision-making and social distance, $F(2,54) = 1.38$, $p = .259$. We further performed an ANOVA on the relative frequency of optimal choices and found no main effect for social distance, $F(1,27) = 0.43$, $p = .52$, but a significant interaction effect between level of decision-making and social distance, $F(2,54) = 5.52$, $p = .007$, $\eta_p^2 = 0.17$ (for descriptive statistics see Table 1 in the manuscript).
